# Effectiveness of a Mobile Phone App for Adults That Uses Physical Activity as a Tool to Manage Cigarette Craving After Smoking Cessation: A Study Protocol for a Randomized Controlled Trial

**DOI:** 10.2196/resprot.4600

**Published:** 2015-10-22

**Authors:** Mary Hassandra, Taru Lintunen, Tarja Kettunen, Mauno Vanhala, Hanna-Mari Toivonen, Kimmo Kinnunen, Risto Heikkinen

**Affiliations:** ^1^ Department of Sport Sciences University of Jyväskylä Jyväskylä Finland; ^2^ Department of Health Sciences University of Jyväskylä Jyväskylä Finland; ^3^ Central Finland Central Hospital Jyväskylä Finland; ^4^ Unit of Family Practice Central Hospital of Central Finland University of Eastern Finland Kuopio Finland; ^5^ Agora Center University of Jyväskylä Jyväskylä Finland; ^6^ Statistical Analysis Services Analyysitoimisto Statisti Oy Jyväskylä Finland

**Keywords:** behavior change, mHealth app, physical activity, randomized controlled trial, relapse prevention, smoking

## Abstract

**Background:**

Results from studies on the effects of exercise on smoking-related variables have provided strong evidence that physical activity acutely reduces cigarette cravings. Mobile technology may provide some valuable tools to move from explanatory randomized controlled trials to pragmatic randomized controlled trials by testing the acute effectiveness of exercise on quitters under real-life conditions. An mHealth app was developed to be used as a support tool for quitters to manage their cigarette cravings.

**Objective:**

The primary aim of this paper is to present the protocol of a study examining the effectiveness of the Physical over smoking app (Ph.o.S) by comparing the point prevalence abstinence rate of a group of users to a comparator group during a 6-month follow-up period.

**Methods:**

After initial Web-based screening, eligible participants are recruited to attend a smoking cessation program for 3 weeks to set a quit smoking date. Fifty participants who succeed in quitting will be randomly allocated to the comparator and experimental groups. Both groups will separately have 1 more counseling session on how to manage cravings. In this fourth session, the only difference in treatment between the groups is that the experimental group will have an extra 10-15 minutes of guidance on how to use the fully automated Ph.o.S app to manage cravings during the follow-up period. Data will be collected at baseline, as well as before and after the quit day, and follow-up Web-based measures will be collected for a period of 6 months. The primary efficacy outcome is the 7-day point prevalence abstinence rate, and secondary efficacy outcomes are number of relapses and cravings, self-efficacy of being aware of craving experience, self-efficacy in managing cravings, and power of control in managing cravings.

**Results:**

Recruitment for this project commenced in December 2014, and proceeded until May 2015. Follow-up data collection has commenced and will be completed by the end of December 2015.

**Conclusions:**

If the Ph.o.S app is shown to be effective, the study will provide evidence for the use of the app as a support tool for people who are trying to manage cravings during smoking cessation programs. It is anticipated that the results of the study will provide knowledge of how physical activity affects cigarette craving in real-life situations and inform the development and delivery of relapse prevention in smoking cessation treatment.

**Trial Registration:**

International Standard Randomized Controlled Trial Number (ISRCTN): ISRCTN55259451; http://www.controlled-trials.com/ISRCTN55259451 (Archived by WebCite at http://www.webcitation.org/6cKF2mzEI)

## Introduction

Although smoking cessation treatments such as behavioral support, nicotine replacement therapy (NRT), and pharmacological treatment have been effective in the short term, the long-term relapse rates are high. The percentages of relapse are between 95% for the cold turkey method and 75% for combined therapies of counseling and pharmacotherapy [1-3]. A systematic review of the effectiveness of smoking relapse prevention [4] indicates that the most effective long-term methods in preventing relapses are self-help materials. Different types of pharmacotherapy showed varied results from short-term to long-term effectiveness and behavioral interventions for relapse prevention appeared effective in the short-term only. The skills approach to behavioral intervention has been widely studied as a method with promising results [5]. Nevertheless, there is no strong evidence to support any specific behavioral intervention as being most helpful to avoid relapses after quitting smoking and there is little research available regarding other behavioral approaches [6]. The latest efforts to develop a taxonomy of behavior change techniques, used in interventions in general [7] and more specificly in smoking cessation interventions [8-10], are expected to help develop stronger evidence in future research in this field.

Exercise has recently been incorporated as a smoking cessation aid into existing programs with promising results. In general, studies examining the effects of exercise on variables related to smoking cessation have focused on either the long-term effects after long interventions or on the acute effects of exercise on smoking-related variables. A recent review [11] of exercise interventions for smoking cessation revealed mixed results. The heterogeneity of the studies reviewed did not allow credible conclusions to be drawn. They focused more on studies that provided data for long-term effects (ie, follow-up data collection at more than 6 months). Within that focus, they identified 15 trials, 3 of which showed significantly higher abstinence rates in a physically active group versus a control group at the end of treatment [12-14]. One of the studies also showed a significant benefit for exercise versus control on abstinence at the 3-month follow-up and a benefit for exercise at the 12-month follow-up [13].

The main reason for relapsing is the craving that smokers experience. Results on the effects of exercise on smoking-related variables have provided strong evidence that physical activity acutely reduces cigarette craving [15-17]. Fourteen reviewed studies that compared a bout of exercise with a passive condition or compared 2 intensities of exercise concluded that even relatively small doses of exercise should be recommended as an aid to managing cigarette cravings and withdrawal symptoms [15]. The results of 12 reviewed studies were similar, showing that cigarette craving was reduced following exercise with a wide range of intensities, from isometric exercise and yoga to activities with heart rates as high as 80%-85% [16]. Using individual participants’ data from 17 studies, participants’ engagement in physical activity was compared to that of control group participants. The results showed that the effects of all primary studies were in the same direction, with physical activity groups showing a greater reduction in cravings compared with controls, implying strong evidence that physical activity acutely reduces cigarette craving [17].

Evidence of the positive effects of exercise on acute reduction of cravings from experimental laboratory-based studies is strong [15-18], but findings are limited by a lack of long follow-up and artificial settings. Therefore, there is a need to start experimenting with interventions in real-life situations. One effort in this direction is a recent study that used a lab-based scenario with high ecological validity to show that an acute bout of exercise can reduce cravings following concurrent stressors [19]. Similar attempts to collect data from real life for smoking and exercise behavior using ecological momentary assessment methods (EMAs) have also been observed in the recent literature [20-21]. However, the investigation of the effects of short bouts of exercise as a means to acutely manage cravings in real life and the long-term effects of this method on relapse rates has not yet been the focus of any study. Our approach is similar to EMA methods in that it offers real-time data, but it differs in that entries are self-initiated and in how the app also acts as an intervention by offering suggestions to overcome craving and supportive motivation messages. The growing use of mobile technology may provide some valuable tools to move from explanatory randomized control trials that test the acute efficacy of exercise on abstinent smokers under highly controlled conditions to pragmatic randomized control trials that test the acute effectiveness of exercise on quitters under real-life conditions.

Mobile technologies are a means for providing individual-level support to health care users and a promising platform for health interventions [22]. Behavioral intervention technologies offer promising opportunities to expand psychological practice [23]. Several mobile health interventions have been designed to increase healthy behavior (eg, to promote smoking cessation or increase activity levels) and are also cost effective [24]. A review of mobile phone-based interventions for smoking cessation revealed only 4 trials: 2 using SMS text messages and 2 using the Internet and mobile phones. The trials were heterogeneous. However, when the data from both programs were pooled, they found statistically significant increases in both short- and long-term self-reported quitting [25]. A more recent systematic review [26] concluded that SMS-based smoking cessation interventions more than doubled biochemically verified smoking cessation at 6 months. Moreover, the self-help methods have been indicated in previous literature as effective for long-term effects [4] and the use of mobile phone applications as a self-help method sounds promising.

Therefore, a mobile phone application has been developed for the needs of this study. The aim of the app is to support quitters in managing their cigarette cravings and abstaining over the long term by using exercise as the main behavioral substitution strategy. The mHealth app called “Physical over Smoking (Ph.o.S)” includes a data collection mechanism to collect real-time data regarding relapses. The Ph.o.S app was developed based on evidence-based practices for relapse prevention after smoking cessation. Following that, a trial has been designed to test the effectiveness of the mHealth app.

### Trial Aim

The aim of this study is to present the protocol of a study that assesses the effectiveness of the Ph.o.S app, which helps abstaining users to manage their cigarette cravings. The study will examine the quit rates of abstaining users of the app versus the quit rates of a comparator group for a 6-month period after quitting smoking. The trial’s main hypothesis is that users of the Ph.o.S app will have higher 7-day point prevalence abstinence rates during the follow-up measures in comparison to the comparator group (trial registration number ISRCTN55259451).

Four additional hypotheses have been proposed:

1. Users of the Ph.o.S app will have fewer self-reported relapses during the follow-up measures than the comparator group will.
2. Users of the Ph.o.S app will report higher self-efficacy from being aware of experiencing cravings than the comparator group will.
3. Users of the Ph.o.S app will report higher self-efficacy in managing cravings in relapse situations than the comparator group will.
4. Users of the Ph.o.S app will report a higher power of control to manage cravings in everyday situations than the comparator group will.

### Trial Design

This study is a 2-armed randomized clinical trial. Participants identified as eligible and who give their consent to participate will receive a smoking cessation program consisting of 3 group sessions (once per week). After the quit day, participants will be randomly assigned to 2 groups. Both groups will have a separate fourth session where they will receive training on relapse prevention and a plan to cope with cravings. The intervention group will receive an additional short training session on how to use the Ph.o.S app as an additional support tool whenever they experience cravings in their everyday life during the follow-up period. Preintervention, postintervention, and 6-month follow-up assessments will be conducted. Table 1 displays an overview of the study’s timeline and the schedule of enrollment, interventions, and assessments. Multimedia Appendix 1 displays the detailed measurement time points for each group.

**Table 1:** Study timeline: overview of schedule of enrollment, interventions, and assessments.

Timeline points	Schedule				
	Screening for eligibility	Intervention: quit smoking (3 sessions)	Quit day: allocation	Intervention: manage cravings (4th session)	Follow-up assessment
-*t*_3_	X				
-*t*_2_		X			
-*t*_1_		X			
*t*_0_			X		
*t*_1_				X	
*t*_2_ (3 days after)					X
*t*_3_ (1 weeks after)					X
*t*_4_ (2 weeks after)					X
*t*_5_ (3 weeks after)					X
*t*_6_ (4 weeks after)					X
*t*_7_ (12 weeks after)					X
*t*_8_ (24 weeks after)					X

## Methods

### Study Setting

The study setting is the Jyväskylä Community Primary Health Care Center in Central Finland.

### Eligibility Criteria

All interested participants are screened for eligibility by completing a short battery of questions online. Noneligible participants are advised to contact their doctor or nurse for help. Participants are adults (between the ages of 18 and 65) who have been smokers for at least 1 year and who smoke more than 10 cigarettes per day. (Those who use snus only are excluded.) They should have no other addictions (ie, alcohol, prescription drugs, or illegal drugs) according to the behavioral screening tool NIDA Quick Screen V1.0 [27]. Eligible participants should be addicted to nicotine, with a score of more than 4 out of 10 on the Tobacco Dependence Screener (TDS) [28]. TDS is a 10-item self-report questionnaire, and it generates a continuous dependence score. It demonstrates acceptable reliability in different samples as well as validity because it has a high association with smoking heaviness measures (eg, number of cigarettes smoked per day, carbon monoxide levels) and years of smoking [28-29]. Participants should have a strong motivation to quit and score more than 3 out of 7 on the Motivation to Stop Smoking Scale (MTSS) [30]. The MTSS is a single-item scale assessing an individual’s motivation to quit smoking, combining aspects of desire and intention to quit. Scores range from 1 (I don’t want to stop smoking) to 7 (I really want to stop smoking and intend to in the next month). Participants are also screened for active psychological distress by completing the 12-item General Health Questionnaire [31] and those scoring more than 20 out of 36 are excluded [32,33] because they have lower quit rates [34,35]. Participants are screened for health risks if they increase their physical activity by completing the Physical Activity Readiness Questionnaire [36]. (Those answering yes to any question will be excluded.) Nevertheless, participants who were able to provide permission to exercise from their doctor were accepted to the program. For example, if their blood pressure, heart condition, or bone or joint problem is well controlled with drugs and their doctor says it is safe to exercise.

Finally, the Gold Standard Monitoring Form used by the National Health Service in the United Kingdom is completed for each participant [37]. The form records useful information regarding participants’ personal details, information about the type of intervention, any pharmacological support (eg, NRT, varenicline), use of other nicotine products (eg, snus), and the outcome. Participants currently trying to quit or taking a smoking cessation medication (eg, NRT, wellbutrin, or varenicline) or receiving any other form of treatment (eg, Web-based, app-based, or other) are not excluded as long as they agree to follow our methods.

### Recruitment, Randomization, and Allocation

Recruitment will occur through referrals from the health care units in the Jyväskylä area as well as via the Web pages of Central Finland Respiratory Association, the City of Jyväskylä, Radio Jyväskylä, and Yle News. All interested participants will be screened for eligibility. The enrollment period ended in May 2015. The first 50 eligible participants will start the smoking cessation intervention, which consists of 3 weekly counseling sessions and will help them set a quit date. The rest of the eligible participants will be put on a waiting list and invited to participate in case of dropouts before the quit day/randomization time point. The first 50 participants to reach the quit day/randomization time point will then form the intention-to-treat group that will continue in the study. After the quit day/randomization, participants will be assigned to an experimental group (25 participants) and a comparator group (25 participants), and both groups will separately have 1 more counseling session on how to manage cravings. In this fourth session, the only difference in treatment between the groups is that the experimental group will have an extra 10-15 minutes of guidance on how to use the Ph.o.S app to manage cravings during the follow-up period. Both groups will be followed up for the main and secondary efficacy outcomes of interest for 6 months. The method of randomization is a set of numbers generated by online software [38]. The principal investigator will generate the allocation sequence and the study nurse will assign participants to the groups. The study nurse is blind to the allocation sequence until the third session and the principal investigator is blind to the participants and their data up to the first follow-up measure.

### Interventions

#### Smoking Cessation and Relapse Prevention Interventions

The presentation of the smoking cessation intervention content has been based mainly on the taxonomy of behavior change techniques in interventions [7] and the taxonomy of behavior change techniques used as support for smoking cessation [39]. The theoretical frameworks that the techniques are based on are control theory [40], the social-cognitive theory [41], the theory of planned behavior [42], and the motivational interviewing technique [43]. During the first session, baseline data are collected and then a group or individual motivational interview session takes place for about an hour, aiming to motivate participants to take further responsibility for making important health-related behavior changes (ie, to gradually decrease smoking and be more physically active). During the second session, participants discuss the barriers for behavior change and they brainstorm facilitators to apply. In the third session they set specific goals and make plans for behavior change. In this meeting, participants also set a quit day and they get training on the use of pedometers. The 3 sessions are scheduled weekly. The intervention is a shorter version of a previously effective intervention named “No more smoking! It’s time for physical activity” [44-45]. Multimedia Appendix 2 contains a more detailed overview of the contents of the intervention used to help participants quit smoking.

All sessions are delivered by the study nurse, who has previous experience with counseling smokers to quit. Multimedia Appendix 3 presents the content of the fourth session that covers relapse prevention, which is held for the participants within 1 week after their quit day. The aim of this fourth session is to help participants develop a cravings management plan to prevent relapses. Similarly, the presentation of the fourth session’s content has been based on the taxonomy of behavior change techniques used as support for smoking cessation [39] and in general interventions [7]. The theoretical frameworks that the techniques are grounded in are the relapse prevention model [46,47], control theory [40], social-cognitive theory [41], and the theory of planned behavior [42].

#### Database and Data Collection Mechanism of the Ph.o.S App

The mHealth mobile phone application, Ph.o.S, and its data collection mechanism was developed by a software designer affiliated with the University of Jyvaskyla, as a project researcher at Agora Center. Additional support for the development was provided for graphic design and user interface design by 2 students from the IT department. Table 2 presents examples of the database contents by category (eg, introductory messages, physical activities, and motivational messages). The database contents are based on the relapse prevention model [46,47] and the behavior change techniques for smoking cessation via text-messaging intervention [10]. Additionally, the Ph.o.S app was designed according to the principles of persuasive systems design [48]. For example, the principles of tailoring [49], personalization, suggestion, praise, trustworthiness, and expertise have been used in the design of the application. The application database includes a pool of 57 introductory messages, 49 motivational messages, and 64 physical activities, all of which are coded to appear according to the users’ profile and status. Introductory and motivational messages were reviewed by 3 experts on health and exercise psychology and physical activities by 2 experts on sport and exercise science. Multimedia Appendix 4 includes examples of screenshots of the sequence from a sample usage session.

During the fourth session, experimental group participants are instructed to use the application whenever they experience cigarette cravings and to give feedback for every use. As a result, data entries are self-initiated whenever participants use the app. The Ph.o.S. app is installed on participants’ personal mobile phones, but if they do not have a mobile phone, then a device is provided by the study team. No incentives are provided to use the app or to complete data during the entire follow-up assessment period. When they need to, participants can use the app without an Internet connection. All information is uploaded to the server as soon as the device is connected. Participants’ identification number and profile settings are entered when the Ph.o.S app is installed on their mobile phones and used for the first time. Profile settings include gender, age, weight, and height, days since quit date, the origin of the decision to quit, and heavy and moderate intensity physical activity history during a typical week (screenshot 1, Multimedia Appendix 4). After that, every time a user experiences a craving situation, specific information regarding mood (eg, positive, neutral, negative), place (eg, home, at work, outdoors), and social environment (eg, alone, not alone) are requested (screenshot 2, Multimedia Appendix 4). According to users’ profile information and the situational status, a variety of animated physical activities, matched with introductory and motivational messages, are suggested. Physical activities were animated to model/demonstrate the suggested activities in a visual form (screenshot 3, see Multimedia Appendix 4). Each time the participant uses the app, he or she is asked to provide feedback on the effectiveness of the app to manage the craving. Answer options are the following: “Yes it worked,” “No, but I’ll manage,” or “No, I relapsed” (screenshot 4, see Multimedia Appendix 4). Multimedia Appendix 5 shows the CONSORT checklist for this study.

**Table 2:** Ph.o.S app: examples of database content.

Category	Examples	Behavior change techniques
Introductory messages	Researchers have found 70 poisonous chemicals in cigarettes which cause cancer. Stay healthy!	Provide information on consequences of behavior in general
	Your shortness of breath when using stairs is much less now!	Provide information on consequences of behavior to the individual
	Ex-smokers have less than two cravings a day. You can manage this craving!	Provide normative information about others’ behavior
	You are a good example to others every day you stay away from smoking!	Prompt identification as role model/position advocate
	Is it worth giving up what you’ve worked so hard for? Definitely not!	Prompt anticipated regret
	Walk proudly! You are doing an amazing job quitting!	Prompt rewards contingent on effort or progress toward behavior
	
Physical activities	Walking: Walk and every 10 steps say: You can make it!	Behavioral substitution + Prompt self-talk
	Brisk walking: Walk briskly and every 15 steps say: You can do this!	Behavioral substitution + Prompt self-talk
	Stairs: Find a stairway to go up and down!	Behavioral substitution
	Breathing: Inhale for a count of 4, then exhale for a count of 4.	Behavioral substitution
	Tension release: Grab a ball and squeeze it with your right hand and then with your left hand.	Behavioral substitution
	Stretching: Stretch your upper arms. Hold for 10 seconds.	Behavioral substitution
	Balance: Balance on your right leg and then on your left leg for 5 seconds.	Behavioral substitution
	Strength: Do as many push-ups as you can in a row.	Behavioral substitution
	Isometric: Hold the superman position for 5 seconds.	Behavioral substitution
	Dance: Listen to your favorite song and dance!	Behavioral substitution
	Gardening, cleaning home physical activities: Do some housework!	Behavioral substitution
	
Motivational messages	Pain is temporary. Quitting is forever!	Facilitate relapse prevention and coping
	I can keep going!	Enhance self-regulation
	Never be a slave to cigarettes again!	Motivation to remain abstinent
	Do not go back! You are a permanent ex-smoker now!/You are a healthy ex-smoker!	Motivation to remain abstinent
	Concentrate on your goal!	Maintain engagement
	It’s not the situation; it’s your reaction to the situation!	Provide information on withdrawal symptoms
	I want to feel like a winner, not miserable after 3 minutes!	Prompt anticipated regret
	My actions are always within my control.	Self-regulation/self-control

### Participant Retention

**Figure 1:**
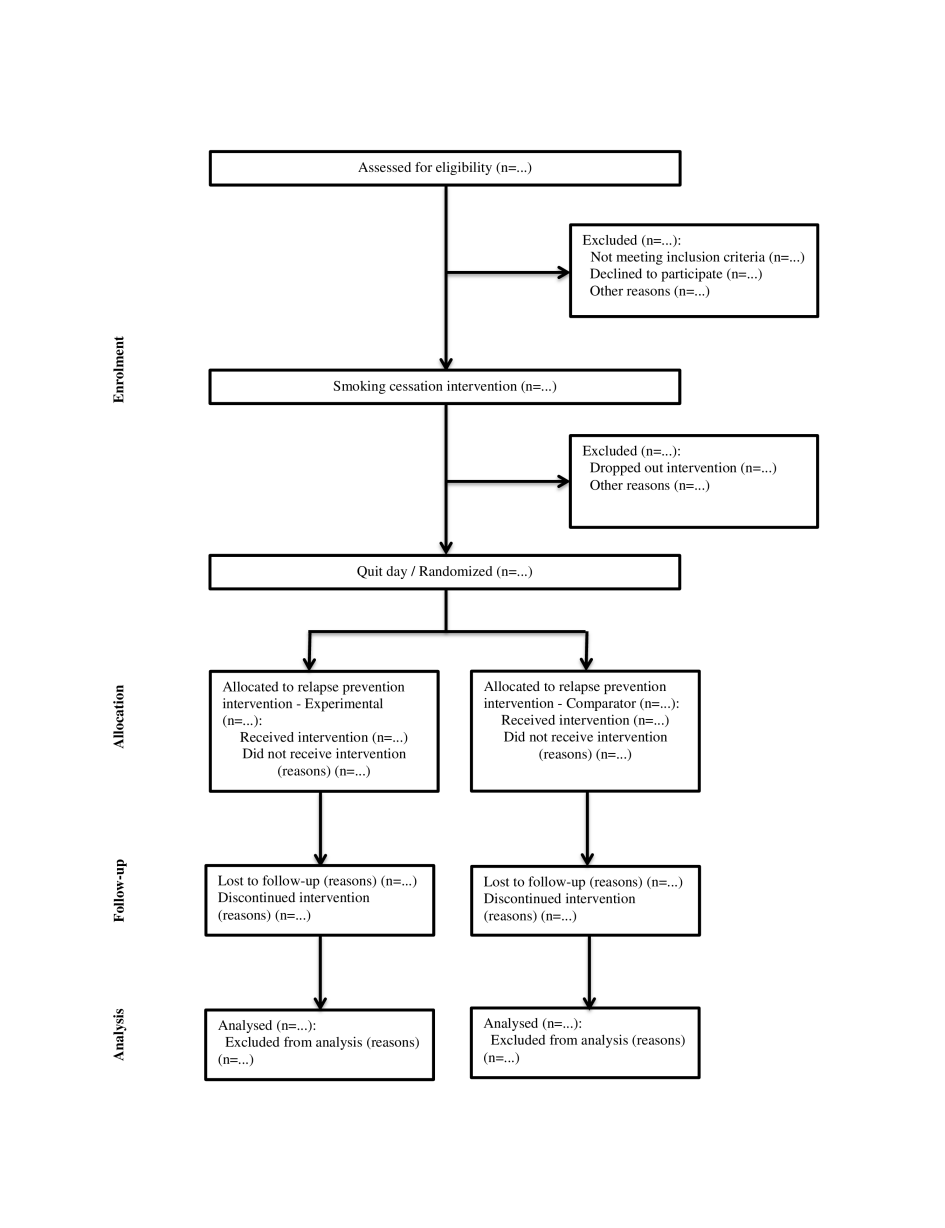
Study flowchart: The diagram illustrates the flow of participants.

To prevent missing data, an effort will be made to retain the participants in the trial for the follow-up data collection. However, participants who withdraw their consent will discontinue their participation in the study. Moreover, participants who attend less than 2 out of 3 sessions of the quit smoking intervention will be excluded from the final analysis. Participants who drop out of the trial or who are lost for follow-up are considered to be smoking. The flow of the participants is presented in Figure 1.

The strategies for monitoring and improving adherence to intervention protocols include the following:

1. Face-to-face adherence reminder. Study participants will be well informed during the first session about the expectations regarding study procedures before they sign the informed consent form. Moreover, a reminder about the expected participation during the follow-up measures takes place during the fourth session when they are asked about their preferred way of follow-up data collection (eg, telephone, mail, email or SMS).
2. Providing feedback about how well subjects adhere to protocol and achieve target goals whenever possible. During the follow-up period a short SMS or email message will be sent to all participants (ie, both experimental and comparator). The follow-up messages start with an acknowledgment of the participant’s contribution to date (ie, how well they adhere to the protocol), followed by a motivational message that is individualized depending on their progress (ie, relapse or no relapse). Next, information on how to fill out the follow-up questionnaire is provided and, finally, the date of the next follow-up point is reported.
3. Monitoring adherence to the Ph.o.S app use and intervening when adherence problems emerge. In the experimental group, adherence is monitored by recording through the data collection mechanism of the Ph.o.S app. When participants do not use the Ph.o.S app for 1 week, they are asked, via SMS or email, to verify if they use the Ph.o.S app, to check if there is a technical problem, or if they have stopped using the Ph.o.S app. If they answer that they do not use the app, then they are sent a link to a short questionnaire in order to assess the reasons for discontinuing use of the app. Participants are considered dropouts when they do not respond to the invitation emails for measurement but not when they stop using the app.

## Results

### Primary Efficacy Measure

Point prevalence abstinence (PPA) will be the primary outcome and the primary efficacy parameter for the study. The main outcome analyses are based on 7-day PPA (ie, reported abstinence of at least 7 days prior to each scheduled follow-up). PPA and prolonged abstinence are closely related and can be interconverted with moderate accuracy [50]. Self-reports of smoking behavior over the last 7 days are collected from participants at the fourth session (*t*_1_), 3 days after the fourth session day (*t*_2_), weekly after the fourth session day through week 4 (*t*_3_-*t*_6_), and at weeks 12 (*t*_7_) and 24 (*t*_8_) after the fourth session day. Self-reported PPA at the *t*_1_ and *t*_8_ follow-ups are verified by saliva cotinine (with a cutoff value of 10 ng/mL) for stated abstinence using NicAlert saliva-cotinine tests [51]. The timeline follow-back for smoking is used to assess daily smoking intensity [52] in a small 1-week calendar form on paper to record how many cigarettes they smoked each day in the last 7 days.

### Secondary Efficacy Measures

The secondary efficacy parameters for the study include the following measures:

1. The self-reported number of relapses, which is assessed through a single item asking participants to complete the number of relapses they had the last 7 days. Data are collected from participants at the fourth session (*t*_1_), 3 days after *t*_1_ (*t*_2_), weekly from *t*_1_ through week 4 (*t*_3_-*t*_6_), and at weeks 12 (*t*_7_) and 24 (*t*_8_) after *t*_1_.
2. The self-reported number of cravings, which is assessed through a single item asking participants to complete the number of cravings they experienced in the last 7 days. Data are collected from participants at the fourth session (*t*_1_), 3 days after *t*_1_ (*t*_2_), weekly from *t*_1_ through week 4 (*t*_3_-*t*_6_), and at weeks 12 (*t*_7_) and 24 (*t*_8_) after *t*_1_.
3. Self-efficacy on being aware of experienced cravings, which is assessed with the single item: “How well are you aware of your cigarette cravings?” Answers are given on a 10-point scale from 1 (very poorly) to 10 (very well). Data are collected from participants before the quit day (-*t*_2_), at *t*_1_, *t*_2_, weekly from quit day at *t*_3_-*t*_6_, and at weeks *t*_7_ and *t*_8_.
4. Self-efficacy on managing cravings, which is assessed with the single item: “How well do you manage your cravings?” Answers are given on a 10-point scale from 1 (very poorly) to 10 (very well). Data are collected from participants at -*t*_2_, *t*_1_, *t*_2_, weekly at *t*_3_-*t*_6_, and at weeks *t*_7_ and *t*_8_.
5. Power of control in managing cravings is assessed with 6 items. An example item is “If I am in a situation where I celebrate with my friends...,” then, the answer for the experimental group is “It will be more difficult to use Ph.o.S. app to control my craving for tobacco,” and for the comparator group is “It will be more difficult to do something to control my craving for tobacco.” Answers are given on a 7-point scale from 1 (totally agree) to 7 (totally disagree). The other items present similar tempting situations like when participants are stressed or angry. Data are collected from participants before the quit day at -t_2_ and -t_1_ and after *t*_1_ at weeks t_2_, t_6_, t_7_, and t_8_.

### Additional Measures

Additional measures of mainly psychological variables will be collected in order to use them as possible explanatory variables of the primary or secondary efficacy measures. Moreover, usability, fidelity, and data extracted from the phone database will be collected for validation purposes.

1. Physical activity behavior. Self-reported data regarding participants’ current physical activity behavior as recorded with the International Physical Ativity Questionaire (IPAQ) [53] will be collected at -*t*_2_ and -*t*_1_ and at weeks *t*_7_ and *t*_8_. Self-reported physical activity behavior through self-reports, before the quit day at -*t*_2_ and -*t*_1_, will be verified by measuring their step counts (via pedometer Omron HJ-152) the last week before measurement day. IPAQ records the physical activity of 4 intensity levels for the last 7 days: (1) vigorous-intensity activities such as aerobics, (2) moderate-intensity activities such as leisure cycling, (3) walking, and (4) sitting.
2. Relapse situation efficacy as recorded with the Relapse Situation Efficacy Questionnaire (RSEQ) [54] will be assessed before the quit day at -*t*_2_ and -*t*_1_. RSEQ consists of 75 simple items assessing participants’ confidence in their ability to resist the temptation to smoke in a wide variety of contexts. A 4-point response scale is used from 1(not at all confident) to 4 (extremely confident).
3. Attitude toward, intention to, and perceived behavioral control of increasing physical activity behavior will be measured through a self-reported questionnaire before and after quit day at -*t*_2_ and *t*_1_. Similarly, attitude toward, intention to, and perceived behavioral control of quitting smoking will be measured before quit day at -*t*_2_. Likewise, attitude toward, intention to, and perceived behavioral control of craving management will be measured during the fourth session at *t*_1_. Attitude toward all 3 behaviors (ie, increasing physical activity, quitting smoking, and managing cravings) is assessed with 6 items using a 7-point semantic differential scale (ranging from -3 to +3): ‘‘To (behavior) in the following month will for me be...’’: good-bad, unpleasant-pleasant, wise-silly, easy-difficult, healthy-unhealthy, important-not important. Similarly, intention is measured with 3 items: “In the following month...”: (1) “I intend to (behavior),” (2) “I will try to (behavior),” (3) “I plan to (behavior).” Answers are given on a 7-point scale ranging from 1 (very likely) to 7 (very unlikely). Correspondingly, perceived behavioral control is measured with 4 items, for example: “For me to (behavior) in the next month is...,” and rated on a 7-point scale, with participants answering from 1 (easy) to 7 (difficult).
4. Usability of the Ph.o.S app for the experimental group only will be collected through the System Usability Scale (SUS) self-reported questionnaire [55] 1 week (*t*_3_) and 1 month (*t*_6_) after participants have started using it. SUS is a 10-item scale giving a global view of subjective assessments of usability. Answers are given on a 5-point scale from 1 (strongly agree) to 5 (strongly disagree). Moreover, a follow-up qualitative assessment when the user stops using the app is performed in order to determine the reasons for discontinuation and possible other means they use to manage cravings effectively.
5. All participants are asked fidelity check questions during the follow-up measure at *t*_2_, *t*_3_, *t*_6_, *t*_7_, and *t*_8_. Questions are related to additional help (eg, pharmacological, mHealth apps) used to manage cigarette cravings. Additional questions for the experimental group are related to the ways they use the Ph.o.S app.
6. Information from the Ph.o.S app is also collected constantly during the follow-up period for the experimental group from the time they start using it (*t*_2_-*t*_8_). Data, such as frequency of use and frequency of successful and unsuccessful efforts to manage craving through the suggested solutions, will be extracted and used to verify the corresponding self-reported questions. Additional data will be extracted for descriptive purposes.

### Data Collection Procedure

Screening data at time point -*t*_3_ will be collected via an online questionnaire to determine eligibility for all participants who respond via the various recruitment strategies. The questionnaire consists of a short overview of the aim of the study and questions regarding the person’s addictions, quitting history, willingness to quit, general health, and readiness for physical activity. The website used to assess eligible participants is a safe and secure site called Fluid Surveys. People who are eligible, based on the online assessment, receive a follow-up telephone call from the study nurse to verify eligibility and will be invited to come to the health center to complete the consent to participate and the baseline measures.

Baseline data and behavioral and psychological self-reported data at time points -*t*_2_, -*t*_1_, and *t*_1_ will be collected during the first, third, and fourth sessions of the smoking cessation intervention in a paper-and-pencil format, administered by the study nurse in a quiet area of the unit. Follow-up data at all follow-up time points (*t*_2_-*t*_8_) will be collected via online questionnaires after an invitation from the research assistant to participants, via email or SMS, to complete the questionnaires. In addition, data from the phone app users (experimental group only) will be collected during the follow-up period. Users’ phone data will be uploaded daily to a secure university server reserved for this purpose.

Once participants have participated in the final meeting, the researchers will make every reasonable effort to follow the participant for the entire follow-up period of 6 months. Participants may, however, withdraw from the study for any reason at any time. However, early discontinuation of Ph.o.S app for any reason is not a reason for exclusion from the study.

### Data Management

The University of Jyväskylä is responsible for storing and protecting the research data. The research registry is kept at the University of Jyväskylä in a locked cabinet. All electronic data will be stored on a university computer with password protection. Only the principal investigator and the research assistant will have access to the computer-based data. All participants will be assigned a code number in order to protect their confidentiality. All stored data will be under the unique code number. All data will be entered electronically. Original study forms will be entered and kept on file at the participating site. Participant files are stored in numerical order and in a secure and accessible place and manner. Participant files will be stored for a period of 3 years after completion of the study.

### Statistical Methods

Generalized linear mixed models will be used to examine differences between the groups on the dichotomous primary outcome variable: abstinent versus smoker status at the 6-month follow-up time point (ie, *t*_8_). The differences at all follow-up time points will also be examined, but the 6-month follow-up is considered to be the primary focus. Descriptive statistics will be used to report process data. Any covariates identified in the preliminary analyses will be added to the model. Linear mixed models will be used to analyze the secondary efficacy measures for data that are normally distributed or are approximate to a normal distribution (via data transformations). For count variables, a generalized Poisson mixed model will be applied. Alternatively, we will use nonparametric tests by time point separately.

Finally, 3 separate logistic regressions on the primary dichotomous outcome (abstinent-smoker) will be performed using the theory of planned behavior (TPB) constructs of quit smoking behavior and increased physical activity behavior at the -*t*_2_ time point and the TPB constructs of manage cravings behavior at the *t*_1_ time point as predictors. Linear regressions for the continuous variables of the secondary efficacy outcomes will be used to test the predictive ability of all TPB constructs. All analyses will be conducted using intent-to-treat principles with participants remaining in their originally assigned groups after randomization regardless of adherence or protocol deviation. This is done to increase the likelihood that group differences are due to the intervention and unaffected by biases that lead to an overestimation of the intervention effect [56].

### Power

We determined the power for our primary hypothesis, which stated that the 7-day PPA at the 6-month follow-up would be higher in the experimental group than in the comparator group. In order to detect a difference of 10% in quit rates between the experimental and comparator groups, with an expected quit rate of 25% in the comparator group, the study would require, at 80% power with a two-sided *p* value of 0.05, a sample size of 326 participants. Given the budget and time limitations as well as the location (eg, a small city in a sparsely populated area, which makes recruitment a challenge), a sample size of 50 has been set by the research group as a more realistic number when taking into account the available resources. Given the sample size of 50 participants equally assigned to the groups, we calculated the smallest PPA that we could detect with power greater than 0.80. Assuming that the abstinence in the comparator group will be 25% [1-3], power analysis through online calculations [57] indicate that we will be able to detect a significant difference between the experimental and comparator groups with power greater than 0.80 if the PPA in the experimental group is 59% or higher. The percentage of 59% required to obtain statistical power is considered to be very high. At the end of the study we will re-estimate the power of the final percentage differences between groups in the effectiveness report, depending on the exact final sample size as well. Nevertheless, the sample size is considered as an a priori limitation to the study.

### Ethics and Dissemination

Ethics approval has been obtained from the Ethics Committee of the Central Finland Health Care District (Keski-Suomen Sairaanhoitopiirin Eettinen Toimikunta).

### Consent

All eligible participants are given a copy of the information sheet and informed consent form to read. The information sheet provides a summary of the research study and the informed consent document states what the individual is about to participate in, the individual’s rights as a research participant, and information about confidentiality. The study nurse explains all aspects of the study and answers all of the participant’s questions regarding the study. If the person chooses to participate in the study, that person will be asked to sign the informed consent form. No study procedure is performed prior to signing the informed consent form. Subjects who refuse to participate or who withdraw from the study are treated without prejudice. The reason for refusal or withdrawal will be noted on the form if reported.

### Confidentiality

All study-related information will be stored securely at the study site under a coded identification number in order to maintain participant confidentiality. All records that contain names or other personal identifiers, such as screening data for eligibility and informed consent forms, will be stored separately from study records identified by code number. All local databases are secured with password-protected access systems. Forms, lists, logbooks, appointment books, and any other listings that link participant identification numbers to other identifying information will be stored in a separate, locked file in an area with limited access. Participants’ study information will not be disclosed to third parties.

## Discussion

This trial and its findings will contribute to the evidence available to inform the development and delivery of relapse prevention on smoking cessation treatment. The primary objective of the study is to contribute to the literature calling for the development of mHealth applications that support individuals in remaining abstinent after they quit smoking. The overall implementation of the project can have an impact on participants’ motivation to initiate and adhere to physical activity. Increasing physical activity can reduce health-risk factors and improve self-esteem as well as quality of life. The use of the Ph.o.S app is not restricted to any specific place, time, situation, or quitting method and it is free to use. It can also contribute to the reduction of health care costs. In addition to the impact on public health, the project will have a significant contribution to future research on how physical activity affects cigarette cravings in real-life situations and will offer the potential to improve the understanding of the mechanisms that underlie these effects. Nevertheless, the relatively small sample size due to time and budget limitations, as well as the recruitment and engagement difficulties that are common with smokers in clinical trials [58], is a threat to the power of the trial.

### Trial Status

Recruitment for this project commenced in December 2014 and proceeded until May 2015. Follow-up data collection has commenced and will be completed by the end of December 2015.

## Appendix

Multimedia Appendix 1 Study timeline: Schedule of enrollment, interventions, and assessments.

Multimedia Appendix 2 Overview of the smoking cessation interventions content (Sessions 1, 2, and 3).

Multimedia Appendix 3 Overview of the relapse prevention intervention content (Session 4).

Multimedia Appendix 4 Ph.o.S. flow: Examples of screenshots.

Multimedia Appendix 5 CONSORT Checklist form.

## Abbreviations

EMA: Ecological Momentary Assessment Methods
IPAQ: International Physical Ativity Questionaire
MTSS: Motivation to Stop Smoking Scale
NRT: nicotine replacement therapy
Ph.o.S: Physical over Smoking app
PPA: point prevalence abstinence
RSEQ: Relapse Situation Efficacy Questionnaire
TDS: Tobacco Dependence Screener
TPB: theory of planned behavior
