# Supplementary material for: Effectiveness of a Mobile Phone App for Adults That Uses Physical Activity as a Tool to Manage Cigarette Craving After Smoking Cessation: A Study Protocol for a Randomized Controlled Trial
Source: JMIR Res Protoc. 2015 Oct 22;4(4):e125. doi: 10.2196/resprot.4600 (PMC4704920; doi:10.2196/resprot.4600)
Supplement: Multimedia Appendix 1 [file resprot_v4i4e125_app1.doc]

|  | **Time points** | | | | | | | | | | | | | | | | | | | | | |
| --- | --- | --- | --- | --- | --- | --- | --- | --- | --- | --- | --- | --- | --- | --- | --- | --- | --- | --- | --- | --- | --- | --- |
|  | ***-t_3_*** | ***-t_2_*** | ***-t_1_*** | ***t_0_*** | ***t_1_*** | | ***t_2_*** (3d. after) | | ***t_3_*** (1w. after) | | ***t_4_*** (2w. after) | | ***t_5_*** (3w. after) | | ***t_6_*** (4w. after) | | ***t_7_*** (12w. after) | | | ***t_8_*** (24w. after) | | |
|  | All | All | All | All | Com | Exp | Com | Exp | Com | Exp | Com | Exp | Com | Exp | Com | Exp | | Com | Exp | | Com | Exp |
| **Screening for eligibility** |  |  |  |  |  |  |  |  |  |  |  |  |  |  |  |  | |  |  | |  |  |
| **Intervention: Quit Smoking (3 sessions)** |  |  |  |  |  |  |  |  |  |  |  |  |  |  |  |  | |  |  | |  |  |
| **Quit day: Allocation** |  |  |  |  |  |  |  |  |  |  |  |  |  |  |  |  | |  |  | |  |  |
| **Intervention to manage cravings (4^th^ session)** |  |  |  |  |  |  |  |  |  |  |  |  |  |  |  |  | |  |  | |  |  |
| **Follow-up** |  |  |  |  |  |  |  |  |  |  |  |  |  |  |  |  | |  |  | |  |  |
| **Assessments** |  |  |  |  |  |  |  |  |  |  |  |  |  |  |  |  | |  |  | |  |  |
| Screening for Drug Use | **x** |  |  |  |  |  |  |  |  |  |  |  |  |  |  |  | |  |  | |  |  |
| Tobacco Dependence Screener | **x** |  |  |  |  |  |  |  |  |  |  |  |  |  |  |  | |  |  | |  |  |
| Motivation to Stop Smoking Scale | **x** |  |  |  |  |  |  |  |  |  |  |  |  |  |  |  | |  |  | |  |  |
| General Health Questionnaire | **x** |  |  |  |  |  |  |  |  |  |  |  |  |  |  |  | |  |  | |  |  |
| Physical Activity Readiness Questionnaire | **x** |  |  |  |  |  |  |  |  |  |  |  |  |  |  |  | |  |  | |  |  |
| Gold Standard Monitoring Form |  | **x** |  |  |  |  |  |  |  |  |  |  |  |  |  |  | |  |  | |  |  |
| Smoking and quitting history |  | **x** |  |  |  |  |  |  |  |  |  |  |  |  |  |  | |  |  | |  |  |
| Smoking behavior 7days |  | **x** | **x** |  | **x** | **x** | **x** | **x** | **x** | **x** | **x** | **x** | **x** | **x** | **x** | **x** | | **x** | **x** | | **x** | **x** |
| International Physical Activity Questionnaire |  | **x** | **x** |  |  |  |  |  |  |  |  |  |  |  |  |  | | **x** | **x** | | **x** | **x** |
| 3 days of step counts |  | **x** | **x** |  |  |  |  |  |  |  |  |  |  |  |  |  | |  |  | |  |  |
| Relapse situation efficacy |  | **x** | **x** |  |  |  |  |  |  |  |  |  |  |  |  |  | |  |  | |  |  |
| Attitude: quit smoking |  | **x** |  |  |  |  |  |  |  |  |  |  |  |  |  |  | |  |  | |  |  |
| Intention: quit smoking |  | **x** |  |  |  |  |  |  |  |  |  |  |  |  |  |  | |  |  | |  |  |
| Perceived behavioral control - quit smoking |  | **x** |  |  |  |  |  |  |  |  |  |  |  |  |  |  | |  |  | |  |  |
| Attitude: physical activity |  | **x** |  |  | **x** | **x** |  |  |  |  |  |  |  |  |  |  | |  |  | |  |  |
| Intention: physical activity |  | **x** |  |  | **x** | **x** |  |  |  |  |  |  |  |  |  |  | |  |  | |  |  |
| Perceived behavioral control: physical activity |  | **x** |  |  | **x** | **x** |  |  |  |  |  |  |  |  |  |  | |  |  | |  |  |
| Attitude: manage cravings |  |  |  |  | **x** | **x** |  |  |  |  |  |  |  |  |  |  | |  |  | |  |  |
| Intention: manage cravings |  |  |  |  | **x** | **x** |  |  |  |  |  |  |  |  |  |  | |  |  | |  |  |
| Perceived behavioral control - manage cravings |  |  |  |  | **x** | **x** |  |  |  |  |  |  |  |  |  |  | |  |  | |  |  |
| Power of control on manage cravings |  | **x** | **x** |  |  |  | **x** | **x** |  |  |  |  |  |  | **x** | **x** | | **x** | **x** | | **x** | **x** |
| Efficacy on being aware of experience cravings |  | **x** |  |  | **x** | **x** | **x** | **x** | **x** | **x** | **x** | **x** | **x** | **x** | **x** | **x** | | **x** | **x** | | **x** | **x** |
| Efficacy on managing cravings |  | **x** |  |  | **x** | **x** | **x** | **x** | **x** | **x** | **x** | **x** | **x** | **x** | **x** | **x** | | **x** | **x** | | **x** | **x** |
| Number of relapses last 7days |  |  |  |  | **x** | **x** | **x** | **x** | **x** | **x** | **x** | **x** | **x** | **x** | **x** | **x** | | **x** | **x** | | **x** | **x** |
| Number of cravings last 7days |  |  |  |  | **x** | **x** | **x** | **x** | **x** | **x** | **x** | **x** | **x** | **x** | **x** | **x** | | **x** | **x** | | **x** | **x** |
| Cotinine in saliva |  |  |  |  | **x** | **x** |  |  |  |  |  |  |  |  |  |  | |  |  | |  | **x** |
| Relapse debriefing form |  |  |  |  |  |  | **x** | **x** | **x** | **x** |  |  |  |  |  |  | |  |  | |  |  |
| Fidelity check (comparator group) mean |  |  |  |  |  |  | **x** |  | **x** |  |  |  |  |  | **x** |  | | **x** |  | | **x** |  |
| Fidelity check (experimental group) |  |  |  |  |  |  |  | **x** |  | **x** |  |  |  |  |  | **x** | |  | **x** | |  | **x** |
| Data from Ph.o.S app collection mechanism |  |  |  |  |  |  |  | **x** |  | **x** |  | **x** |  | **x** |  | **x** | |  | **x** | |  | **x** |
| Ph.o.S app Usability |  |  |  |  |  |  |  |  |  | **x** |  |  |  |  |  | **x** | |  |  | |  |  |
| All = All participants; Com = Comparator group; Exp = Experimental group | | | | | | | | | | | | | | | | | | | | | | |
