# Supplementary material for: Effectiveness of a Mobile Phone App for Adults That Uses Physical Activity as a Tool to Manage Cigarette Craving After Smoking Cessation: A Study Protocol for a Randomized Controlled Trial
Source: JMIR Res Protoc. 2015 Oct 22;4(4):e125. doi: 10.2196/resprot.4600 (PMC4704920; doi:10.2196/resprot.4600)
Supplement: Multimedia Appendix 2 [file resprot_v4i4e125_app2.doc]

| **Contents** | | **Behavior change techniques** |
| --- | --- | --- |
| ***Session 1*** | | |
| Baseline data collection (*-t_2_*) | | |
| Main objective: Increase awareness and motivation | | |
| Introduction | | |
| Main reasons to quit smoking | Motivational interviewing | |
| Unpleasant symptoms of smoking: Dislikes about smoking |  |  |
| Benefits of quitting smoking |  |  |
| Worries regarding quitting smoking |  |  |
| Daily physical activity habits |  |  |
| Physical activity in relation to smoking |  |  |
| Benefits of being physically active |  |  |
| Dissemination and review of physical activity diary form and pedometers to record daily steps | Prompt self-monitoring of physical activity and smoking behavior | |
| Dissemination and review of smoking behavior diary form |  |  |
| Final comments and reminders | | |
| ***Session 2*** | | |
| Main objective: Barrier identification and problem solving | | |
| Introduction: Week review, reflections, discussion. | Prompt review of behavioral diaries | |
| Identify barriers to quitting smoking. Plans to overcome barriers this week  Identify facilitators to quitting smoking. Facilitators to try this week  Identify ways to decrease the number of cigarettes: Ways to try this week | Prompt barrier identification / Prompt intention formation | |
| Identify barriers to be more physically active: Plans to overcome barriers this week  Identify facilitators to be more physically active: Facilitators to try this week  Identify ways to increase daily physical activity: Ways to try this week | Prompt barrier identification / Prompt intention formation | |
| Final comments and reminders | | |
| ***Session 3*** | | |
| Main objective: Behavior and outcome goal setting; action planning | | |
| Introduction: Week review, reflections, discussion | Prompt review of outcome and behavioral goals | |
| Goals for behavior change: Set the quit smoking day  Dissemination, review and complete the form “My quit smoking day form” | Prompt goal setting (behavior) | |
| Dissemination and review of the weekly plan for decreased numbers of smoked cigarettes per day  Dissemination, review and completion of the form “My small steps to decrease the number of cigarettes I smoke” | Prompt goal setting (behavior)  Set graded tasks | |
| Identify problems for decreasing the numbers of smoked cigarettes per day: Plan solution for each problem  Dissemination, review and complete the form “My daily goals to decrease the number of cigarettes I will smoke” & “Problems and solutions plan” | Prompt intention formation / Action planning  Set graded tasks | |
| Plan to deal with the automaticity of smoking  Dissemination, review and complete the form “How to fight automatic smoking” |  |  |
| Dissemination and review of the weekly plan for increasing the daily physical activity and the form “My small steps to increase my physical activity” | Prompt goal setting (behavior)  Set graded tasks | |
| Identify problems for increasing the daily physical activity: Plan solution for each problem  Dissemination, review and complete the form “My daily goals to increase my daily physical activity” & “Problems and solutions plan” | Prompt intention formation / Action planning  Set graded tasks | |
| Final comments and reminders | | |
| Data collection (*-t_1_*) | | |
| **Quit day (*t_0_*)** | | |
