# Supplementary material for: Effectiveness of a Mobile Phone App for Adults That Uses Physical Activity as a Tool to Manage Cigarette Craving After Smoking Cessation: A Study Protocol for a Randomized Controlled Trial
Source: JMIR Res Protoc. 2015 Oct 22;4(4):e125. doi: 10.2196/resprot.4600 (PMC4704920; doi:10.2196/resprot.4600)
Supplement: Multimedia Appendix 3 [file resprot_v4i4e125_app3.doc]

| **Quit day (*t*_0_)** | |
| --- | --- |
| **Contents** | **Behavior change techniques** |
| ***Session 4*** | |
| ***Comparator group*** | |
| Main objective: Development of craving management plan |  |
| Introduction: Review of the week, reflections, discussion | Prompt review of outcome and behavioral goals |
| Identify past experiences of successful quitting / craving management. | Prompting focus on past success |
| Identify regular, everyday situations that are the riskiest for habitual smoking (e.g., smoking with morning coffee, after dinner). | Relapse prevention/coping planning  Barrier identiﬁcation and  problem solving |
| For every situation, identify 4 kinds of activities that could be performed to avoid temptation or to overcome moments of craving.  Dissemination, review and completion of the form “Habitual craving situations” (for comparator group) |  |
| Identify high-risk situations that are the riskiest for smoking (e.g., important or stressful situations, celebrations, being with other smokers). | Relapse prevention/coping planning  Barrier identiﬁcation and  problem solving |
| For every situation, identify 4 kinds of activities that could be performed to avoid temptation or to overcome moments of craving.  Dissemination, review and completion of the form “Risky craving situations” (for comparator group) |  |
| Final comments and reminders | |
| Data collection (*t_1_*) | |
| ***Intervention Group*** | |
| Main objective: Development of craving management plan | |
| Introduction: Review of the week, reflections, discussion | Prompt review of outcome and behavioral goals |
| Identify past experiences of successful quitting / craving management. | Prompting focus on past success |
| Identify regular, everyday situations that are the riskiest for habitual smoking (e.g., smoking with morning coffee, after dinner). | Relapse prevention/coping planning  Barrier identiﬁcation and  problem solving |
| For every situation, identify at least 3 physical activities that can be performed in order to avoid temptation or to overcome moments of craving.  For every situation, identify 1 additional activity other than a physical one in case physical activity is not possible or the Ph.o.S app does not work.  Dissemination, review and completion the form “Habitual craving situations” (for experimental group) |  |
| Identify high-risk situations that are the riskiest for smoking (e.g., important or stressful situations, celebrations, being with other smokers). | Relapse prevention/coping planning  Barrier identiﬁcation and  problem solving |
| For every situation, identify at least 3 physical activities that can be performed in order to avoid temptation or to overcome moments of craving.  For every situation, identify 1 additional activity other than a physical one in case physical activity is not possible or the Ph.o.S app does not work.  Dissemination, review and completion of the form “Risky craving situations” (for experimental group) |  |
| Introduction to the Ph.o.S app. Aim to support the craving management plan with ideas for physical activities through a smartphone app that has been designed especially for quitters’ needs. | Relapse prevention/coping planning through the Ph.o.S app |
| Dissemination, review and practice of the Ph.o.S app | Prompt practice |
| Final comments and reminders | |
| Data collection (*t_1_*) | |
