# Supplementary figures and images for: Effectiveness of a Mobile Phone App for Adults That Uses Physical Activity as a Tool to Manage Cigarette Craving After Smoking Cessation: A Study Protocol for a Randomized Controlled Trial
Source: JMIR Res Protoc. 2015 Oct 22;4(4):e125. doi: 10.2196/resprot.4600 (PMC4704920; doi:10.2196/resprot.4600)

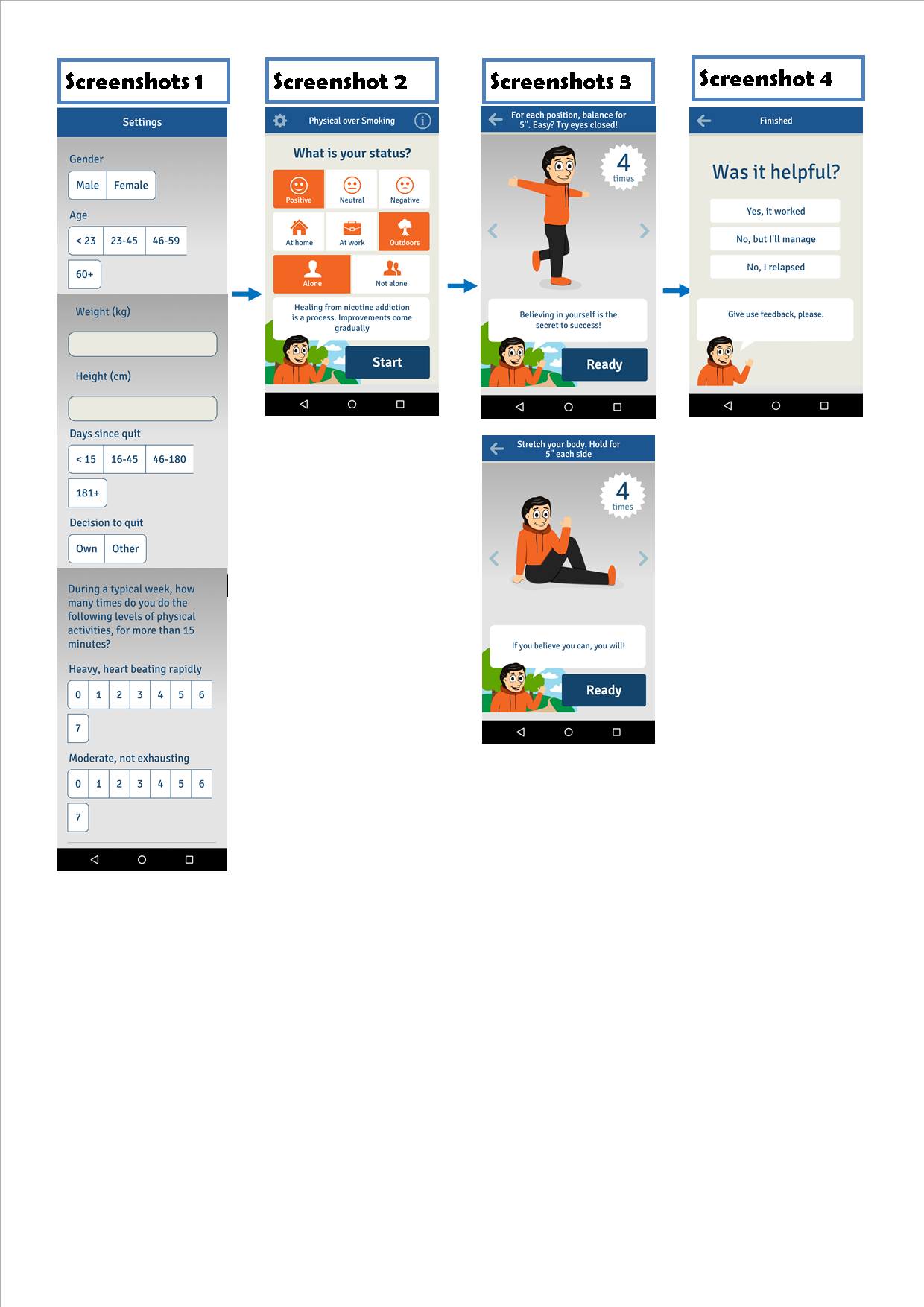

Supplement: Multimedia Appendix 4 [file resprot_v4i4e125_app4.png]
